# Screening for pulmonary tuberculosis in type 2 diabetes elderly: a cross-sectional study in a community hospital

**DOI:** 10.1186/1471-2458-15-3

**Published:** 2015-01-08

**Authors:** Yung-Hsiang Lin, Chia-Pei Chen, Pao-Ying Chen, Jui-Chu Huang, Cheng Ho, Hsu-Huei Weng, Ying-Huang Tsai, Yun-Shing Peng

**Affiliations:** Division of Endocrinology and Metabolism, Department of Internal Medicine, Chang Gung Memorial Hospital, No.6, W. Sec., Jiapu Rd, Puzih City, Chiayi County 613 Taiwan; Department of Diagnostic Radiology, Chang Gung Memorial Hospital, Chiayi, Taiwan; Division of Thoracic and Critical Care Medicine, Chang Gung Memorial Hospital, Chiayi, Taiwan; Chang Gung University, College of Medicine, Tao-Yuan, Taiwan

**Keywords:** Pulmonary tuberculosis, Diabetes mellitus, Risk factors

## Abstract

**Background:**

Tuberculosis is one of the major infectious diseases in Taiwan. It has an especially high prevalence in diabetes patients, in whom it is usually asymptomatic and are more likely to result in drug-resistant tuberculosis. The aim of the study was to aggressively screen high risk diabetic elderly, identify the prevalence of tuberculosis and its determinants.

**Methods:**

Type 2 diabetes patients aged over 65 years were enrolled. They received chest X-rays, blood tests and the questionnaires to assess their medical history and symptoms. Suspicious cases were referred to the pulmonary or infectious disease outpatient clinics. Pulmonary tuberculosis was confirmed by sputum culture. Variables between groups were analyzed by Student *t* test, Chi-square test or Fisher’s exact test. Risk factors were assessed using univariate logistic regression and multiple logistic regression.

**Results:**

A total of 3,087 patients participated this screening program and 7 patients screened positive for pulmonary tuberculosis. Another 5 patients were being under treatment when participating screening program. The prevalence rate was 3.89 per thousand people. The patients with male gender, smoking, liver cirrhosis or subjective body weight loss were associated with an increased risk of tuberculosis significantly. Subjective body weight loss (OR: 6.635 [95% CI: 2.096-21.007]), liver cirrhosis (OR: 10.307 [95% CI: 2.108-50.395]) and history of smoking (OR: 3.981 [95% CI: 1.246-12.718]) are independent risk factors. Among all 73 patients with active tuberculosis or tuberculosis history, they tended to be male, lower body mass index (BMI), more smoking history, more alcohol consumption, more family history of tuberculosis, higher low density lipoprotein (LDL), and less hypertension. However, there was no significant difference in the glycated hemoglobin (HbA1c) levels between the tuberculosis group and non-tuberculosis group.

**Conclusions:**

Active screening program is helpful in detecting pulmonary tuberculosis in elderly diabetes patients. Subjective body weight loss, smoking and liver cirrhosis are independent risk factors.

## Background

Tuberculosis (TB) and diabetes mellitus (DM) are both the major global health problems. According to the World Health Organization (WHO), there were an estimated 8.7 million incident cases of TB and 1.4 million deaths from TB in 2011 [1]. On the other hand, the International Diabetes Federation (IDF) reported that more than 371 million people all over the world had DM and 471 billion USD were spent treating diabetes in 2012 [2]. Many risk factors have been associated with TB, such as male gender [1], cigarette smoking [3, 4], alcohol consumption [5], underweight [6], renal disease [7], household contact [8], socioeconomic status [9], and diabetes mellitus [10]. As diabetes patients increased year by year, more and more studies focused on TB prevention in diabetic group in these years [10–17].

In Taiwan, a total of 12,220 new tuberculosis cases were recorded in 2012, including 8,546 males and 3,674 females [18]. Among these cases, about 52% were aged over 65 years, with the disease possibly induced from impaired immunity with aging. Furthermore, the ratio of extra-pulmonary tuberculosis in Taiwan is decreasing year by year, claiming only about 4% of all incidence tuberculosis in 2010 [19]. Hence, given that they are at high risk for tuberculosis infection, especially pulmonary tuberculosis, it is important for the elderly to be screened. As an aging country composed of more than 11% of the population over 65 years, Taiwan has a high prevalence of diabetes. According to the Nutrition and Health Survey in Taiwan (NAHSIT) 2004–2008 [20], the prevalence of diabetes in Taiwan was 8.8%, higher than the global data of 8.3% [2]. The prevalence was especially high in old people over 65 years, accounting for 27.7% in males and 24% in females, which further increases the risk of tuberculosis infection.

For tuberculosis prevention, many studies focus on the effects of diabetes on tuberculosis infection. The risk of tuberculosis infection not only rises in diabetes patients, but is also associated with a higher risk of an increased number of diabetes complications [10]. Diabetes patients also require longer treatment and are more likely to develop multi-drug resistant tuberculosis [21]. Additionally, joint management of both diseases appears to improve clinical outcomes [17]. However, most studies to date have employed case control or cohort design. In this study, we focus on the active screening of tuberculosis in elderly diabetes patients and assess other possible risk factors among this group of patients.

## Methods

### Study population

This single-center, cross-sectional study was supported by the Centers for Disease Control (CDC) in Taiwan and launched at Chiayi Chang Gung Memorial Hospital (CGMH), which has 1,339 beds and 4 endocrinologists. The selection of study area (Chiayi County) is based on the high proportion of people over 65 years. It reached 16.04% among all administrative districts in Taiwan in 2012 [22]. Meetings were held for the doctors of the hospital and the nurses at the local public health centers who were involved in this study to standardize the screening process before the project.

From 2012 September 1^st^ to November 25^th^, patients with type 2 diabetes mellitus over the age of 65 years were collected from inpatient and outpatient departments of the hospital and 14 of the 16 Public Health Centers of Chiayi county (the other 2 centers had no mobile screening vehicles). Public health nurses were allocated to the mobile units equipped with X-ray machine to actively screen pulmonary TB in diabetic elders in the communities. The study was approved by Chang Gung Medical Foundation Institutional Review Board (No. 101-2656B). Written informed consent was obtained from each participant.

### Data collection

All patients from the hospital and community received screening included a questionnaire, laboratory test, and chest X-ray. The questionnaire was designed to gather basic medical information included age, gender, education, body weight (BW), body height, body mass index (BMI), waist circumference, smoking, alcohol drinking, exercise habit, past history, family history of tuberculosis, and diabetes duration. Symptom selection was based on a 7-point screening method [23], suggested by the WHO and Taiwan CDC, including cough for 2 weeks (score = 2), sputum (score = 2), chest pain (score = 1), poor appetite (score = 1), and subjective body weight loss (score = 1); a total score of 5 or more is suggestive of tuberculosis. The reliability of this method was analyzed in all screening-confirmed tuberculosis patients. Laboratory data included serum fasting sugar, lipid profiles, glycated hemoglobin (HbA1c), renal function, liver function, and urine protein were measured in all patients within 3 months of enrolled date. Chest radiographs within 6 months were also obtained; for hospital cases, they were read by a random radiologist; and for community cases, the X-rays were sent to the Chest Hospital, Department of Health and evaluated by the pulmonologists.

### Confirmation of pulmonary tuberculosis

If tuberculosis was suspected after the screening, the hospital cases were referred to the pulmonary or infectious disease outpatient clinics of CGMH and community cases were referred to the Chronic Disease Center for further evaluation, diagnosis and treatment. Sputum samples were collected from all suspicious cases, one sample per day for three successive days. The samples were sent for acid fast satin and mycobacterial culture, using BACTEC MGIT 960 system and Löwenstein-Jensen solid media simultaneously [24, 25]. Sputum acid fast stain positive was defined as screening positive and sputum culture with Mycobacteria tuberculosis were confirmed cases.

### Statistical analysis

Descriptive statistics are expressed as mean ± SD. All of the continuous variables were detected when the variables followed normal distribution using the Kolmogorov-Smirnov test. Homogeneity of variance was tested with the Levene’s test. Student’s *t*-test applied to evaluate differences in mean values of normally distributed data. Categorical variables were tested using the Chi-square (*χ*^2^) test or Fisher’s exact test. Univariate logistic regression analysis was performed in order to identify potential risk factors associated with pulmonary TB. To control for potential confounders, a multiple logistic regression model with forward selection was used. The entry probability was *p* = 0.1 or less and the removal probability was *p*> 0.2. All statistical tests were two-tailed, and the significance level was set at *p* = 0.05 or less. Model fit was assessed using the Hosmer-Lemeshow goodness of fit test. Data were analyzed using SPSS 18.0 for Windows (SPSS Inc., Chicago, IL, USA).

## Results

### Demographic characteristics

A total of 3,087 (2,141 hospital and 946 community) type 2 diabetes patients over 65 years participated this screening program. The participants without any missing information were 2,979 individuals. The average age of the participants was 74.15 ± 5.90 years old and 62.9% of them were either overweight (BMI 24–27 kg/m^2^) or obese (BMI ≥ 27 kg/m^2^), according to definitions by the Taiwan Ministry of Health and Welfare [26]. About half of the patients had diabetes for longer than 10 years and the average level of HbA1c was 7.47 ± 1.44% (Table 1).

**Table 1:** **Demographic characteristics (n = 3087)**

Variables	Mean ± SD	n (%)
General data		
Age (year)	74.15 ± 5.90	
Gender		
Male		1483 (48)
Female		1604 (52)
BMI (kg/m^2^)		
<24		1146 (37.1)
24-27		1046 (33.9)
≥27		894 (29.0)
missing		1 (0)
DM status		
DM duration		
≥10 years		1573 (51.0)
<10 years		1481 (48.0)
missing		33 (1.1)
Fasting sugar (mg/dL)	146.18 ± 51.79	
HbA1c (%)	7.47 ± 1.44	
HDL (mg/dL)	45.37 ± 12.14	
LDL (mg/dL)	96.84 ± 29.23	
TG (mg/dL)	137.17 ± 89.56	
Creatinine (mg/dL)	1.49 ± 1.56	
ALT (U/L)	29.47 ± 29.20	

Data are n (%) unless indicated.

*Abbreviation*: BMI: body mass index; DM: diabetes mellitus; HbA1c: glycated hemoglobin; HDL: high-density lipoprotein; LDL: low-density lipoprotein; TG: triglyceride, ALT: alanine aminotransferase.

Eleven cases were screening positive (acid fast stain positive) and 7 of them were confirmed with pulmonary tuberculosis (4 non-tuberculosis mycobacteria). Another 5 patients were being under anti-tuberculosis treatment when participating screening program. The TB prevalence rate was 3.89‰ (12/3,087). We defined the cases of screening-confirmed TB and under treatment TB as new TB group (active TB). Another 61 patients had pulmonary tuberculosis history and 2 cases were accidentally confirmed lung cancers.

### Univariate logistic regression analysis for determinants of active pulmonary TB

The difference of clinical characteristics between new TB cases and not-new TB cases are shown in Table 2. Male gender and a history of smoking are statistically significant risk factors of TB infection. Liver cirrhosis was also found to have a higher prevalence in the new TB group.

**Table 2:** **Clinical characteristics of the new TB group and control group**

	New TB	Control	p value	OR (95% CI)
	(n = 12)	(n = 3075)		
**General data**			0.583	1.026 (0.935-1.126)
Age (year)	75.08 ± 3.99	74.14 ± 5.91		
Gender			0.029*	5.438 (1.19-24.859)
Male	10 (83.3)	1473 (47.9)		
Female	2 (16.7)	1602 (52.1)		
BMI (kg/m^2^)	23.81 ± 5.53	25.44 ± 3.86	0.140	0.882 (0.747-1.042)
Smoking			0.009**	4.601 (1.456-14.541)
Yes	7 (58.3)	702 (22.8)		
No	5 (41.7)	2307 (75.0)		
Alcohol consumption			0.274	2.077 (0.560-7.703)
Yes	3 (25.0)	416 (13.5)		
No	9 (75.0)	2592 (84.3)		
**DM status**			0.179	1.006 (0.997-1.014)
Fasting glucose (mg/dL)	166.35 ± 73.91	146.08 ± 51.67		
HbA1c (%)	7.71 ± 1.56	7.46 ± 1.44	0.573	1.112 (0.769-1.608)
HDL (mg/dL)	41.00 ± 10.08	45.38 ± 12.15	0.378	0.966 (0.895-1.043)
LDL (mg/dL)	87.40 ± 20.23	96.86 ± 29.25	0.469	0.988 (0.956-1.021)
Triglyceride (mg/dL)	186.00 ± 143.61	137.02 ± 88.36	0.162	1.002 (0.999-1.006)
Creatinine (mg/dL)	1.39 ± 0.51	1.49 ± 1.56	0.824	0.951 (0.609-1.485)
ALT (U/L)	27.57 ± 12.84	29.48 ± 29.24	0.862	0.997 (0.965-1.030)
DM duration (year)	13.00 ± 9.18	10.61 ± 7.58	0.278	1.037 (0.971-1.106)
**Comorbidities**				
Liver cirrhosis			0.004**	9.710 (2.084-45.24)
Yes	2 (16.7)	62 (2.0)		
No	10 (83.3)	3010 (97.8)		
Hypertension			0.310	0.555 (0.179-1.726)
Yes	6 (50.0)	1975 (64.2)		
No	6 (50.0)	1097 (35.7)		
**Symptoms (Score)**				
Total score ≥ 5			0.765	1.368 (0.176-10.654)
Yes	1 (8.3)	185 (6.0)		
No	11 (91.7)	2784 (90.5)		
Cough ≥ 2 wks (score =2)			0.146	0.218 (0.028-1.694)
Yes	1 (8.3)	872 (28.4)		
No	11 (91.7)	2095 (68.1)		
Sputum (score =2)			0.500	1.513 (0.454-5.039)
Yes	4 (33.3)	737 (24.0)		
No	8 (66.7)	2230 (72.5)		
Chest pain (score =1)			0.994	0.992 (0.128-7.718)
Yes	1 (8.3)	249 (8.1)		
No	11 (91.7)	2718 (88.4)		
Anorexia (score =1)			0.639	1.439 (0.314-6.595)
Yes	2 (16.7)	362 (11.8)		
No	10 (83.3)	2605 (84.7)		
BW loss (score =1)			0.001**	6.997 (2.245-21.809)
Yes	6 (50.0)	371 (12.1)		
No	6 (50.0)	2596 (84.4)		

Data are n (%) unless indicated.

*Abbreviation*: BMI: body mass index; DM: diabetes mellitus; HbA1c: glycated hemoglobin; HDL: high-density lipoprotein; LDL: low-density lipoprotein; ALT: alanine aminotransferase.

New TB group: screening confirmed TB + under treatment TB.

Control group = all participants except new TB.

*p <0.05, **p < 0.01.

A summary of the 7-point symptom screening of all cases is also listed in Table 2. No difference was found for the total score and the ratio of scores above 5 points between the new TB and the not-new TB groups. There was also no statistically significant difference in all symptoms except subjective body weight loss (50% vs. 12.5%, *p* = 0.001).

### Multivariate logistic regression analysis for independent determinants of active pulmonary TB

In multivariate analysis, we included male gender, smoking, liver cirrhosis and body weight loss. Male gender, smoking and liver cirrhosis were the factors that reached statistic significant in univariate analysis in Table 2. The reason why we included body weight loss is that underweight has been shown to be independently associated with tuberculosis [6]. The Hosmer and Lemeshow test indicates a *χ*^2^ of 0.512 (*p* = 0.474), suggesting that the data fit the final model quite well. After adjustment, subjective BW loss (OR: 6.635 [95% CI: 2.096-21.007]), smoking (OR: 3.981 [95% CI: 1.246-12.718]), and liver cirrhosis (OR: 10.307 [95% CI: 2.108-50.395]) remained strongly associated with pulmonary tuberculosis (Table 3).

**Table 3:** **Multiple logistic regression model determining independent risk factors for pulmonary tuberculosis among 3,087 diabetes patients**

Significant variables	Adjust OR	95% CI	p value
Smoking	3.981	1.246-12.718	0.020*
BW loss	6.635	2.096-21.007	0.001**
Liver cirrhosis	10.307	2.108-50.395	0.004**

*Abbreviation*: BW: body weight.

*p <0.05, **p < 0.01.

### The characteristics of all TB group vs. non TB group

To more clearly view the possible risk factors for TB infection, the characteristics of all TB cases (include new TB and TB history cases) and non-TB cases are shown in Table 4. More risk factors achieving statistical significance included male gender, lower BMI, smoking, alcohol drinking, and TB family history. The diabetes control status between the two groups was not significantly different, except for TB patients having a higher low-density lipoprotein (LDL). The prevalence of liver cirrhosis was insignificant in all TB groups. However, hypertension was less frequent in all patients with tuberculosis infection (41% vs. 64.7%, *p*< 0.001).

**Table 4:** **Clinical characteristics of the all TB group vs non TB group**

	All TB	Non TB	p value	OR (95% CI)
	(n = 73)	(n = 3014)		
**General data**				
Age (year)	75.15 ± 5.68	74.12 ± 5.91	0.142	1.029 (0.991-1.069)
Gender			<0.001***	3.663 (2.119-6.333))
Male	56 (76.7)	1427 (47.3)		
Female	17 (23.3)	1587 (52.7)		
BMI (kg/m^2^)	24.37 ± 3.94	25.46 ± 3.87	0.017*	0.923 (0.864-0.986)
Smoking			<0.001***	2.533 (1.576-4.070)
Yes	31 (42.5)	678 (22.5)		
No	41 (56.2)	2271 (75.3)		
Alcohol consumption			0.001**	2.410 (1.425-4.074)
Yes	20 (27.4)	399 (13.2)		
No	53 (72.6)	2548 (84.5)		
TB family history			<0.001***	5.908 (2.437-14.324)
Yes	6 (8.2)	45 (1.5)		
No	67 (91.8)	2969 (98.5)		
**DM status**				
Fasting glucose (mg/dL)	147.15 ± 58.64	146.15 ± 51.62	0.881	1.000 (0.996-1.005)
HbA1c (%)	7.57 ± 1.61	7.46 ± 1.43	0.541	1.050 (0.899-1.226)
HDL (mg/dL)	41.99 ± 11.79	45.44 ± 12.14	0.062	0.974 (0.948-1.001)
LDL (mg/dL)	107.64 ± 35.20	96.60 ± 29.05	0.006*	1.012 (1.003-1.020)
Triglyceride (mg/dL)	138.98 ± 75.20	137.13 ± 89.86	0.886	1.000 (0.997-1.003)
Creatinine (mg/dL)	1.64 ± 1.80	1.49 ± 1.56	0.446	1.054 (0.921-1.206)
ALT (U/L)	27.20 ± 17.82	29.52 ± 29.39	0.615	0.996 (0.982-1.011)
DM duration (year)	10.16 ± 7.37	10.63 ± 7.59	0.606	0.992 (0.960-1.024)
**Comorbidities**				
Liver cirrhosis			0.668	
Yes	2 (2.7)	62 (2.1)		1.340 (0.321-5.585)
No	71 (97.3)	2949 (97.8)		
Hypertension			<0.001***	0.425 (0.266-0.679)
Yes	32 (43.8)	1949 (64.7)		
No	41 (56.2)	1062 (35.2)		

Data are n (%) unless indicated.

*Abbreviation*: BMI: body mass index; DM: diabetes mellitus; HbA1c: glycated hemoglobin; HDL: high-density lipoprotein; LDL: low-density lipoprotein; ALT: alanine aminotransferase.

All TB group: New TB group + patients with TB history.

Non TB group: all participants except all TB group.

*p <0.05, **p < 0.01, ***p < 0.001.

## Discussion

Although diabetes as an important risk factor of tuberculosis infection is well documented [10–13], most of the relevant literature is retrospective. A systemic review in 2010 included only 12 studies about active screening for TB in diabetic patients and some of them used non-specific methods (such as chest X-ray) to diagnose TB [15]. However, screening rate increased with the prevalence of TB in the study region and with the severity of diabetes. One pilot large scale intensive screening project, reported in China [14], enrolled 15,342 diabetes patients over 14 years of age in 5 medical centers without documented HbA1c. Only symptom screening positive patients went on for further studies for TB. From September 2011 to March 2012, the TB notification rate rose from 0.31-1.11‰ (each medical center) to 3.52-7.74‰ (average of 5 centers for each quarter). In a similar symptom screening project in a tertiary care hospital in south India, 38 of 7,083 diabetic patient already had TB and 12 of 125 underwent TB investigation were newly diagnosed TB. The incidence rate increases with HbA1c level and diabetes duration, especially in patients with HbA1c over 9.0% and with diabetes duration over 10 years [16]. Another symptom screening research in Mexico detected 38 TB cases in 7,763 diabetes patients and 11 of them were unaware of their TB [17]. These studies pointed out that active screening and better glucose control might be important in TB prevention.

In Taiwan, there were 12,220 TB notifications in 2012 and 6,328 (52%) of them were over 65 years of age [18]. The prevalence of TB in the general population and in the elderly over 65 years was 0.52‰ and 2.43‰ respectively. In our main study area of Chiayi County, the data was similar, 0.62‰ and 2.54‰, indicating an increased risk of TB infection in the elderly. Similarly, Baker MA et al. reported that diabetes and treated diabetes patients had an increased risk for TB (Hazard Ratio: 3.60 & 4.37, respectively) [10]. According to our findings, the prevalence rate of TB (3.89‰) was further increased 6- to 7-fold in advanced aged diabetes patients, suggesting the worth of wide-spread screening.

Two comorbidities reached statistical significance in our study: higher liver cirrhosis prevalence in the new TB group and lower hypertension prevalence rate in all TB groups. Based on previous research in Taiwan, liver cirrhosis was not found to be a risk factor of pulmonary TB with pulmonary infection [27], but this finding may be due to an insufficient number of active TB cases. Further subgroup studies on the etiologies of liver cirrhosis are also needed to clarify the relation between liver cirrhosis and active tuberculosis in diabetic older patients. Furthermore, BMI was documented to have a positive relationship with blood pressure [28], and being overweight was also associated with high risk of hypertension and cardiovascular events [29]; for these reasons, the lower BMI in all TB groups likely resulted in a lower hypertension prevalence rate. Other risk factors, such as male gender, lower BMI, smoking, alcohol drinking, and TB family history are comparable with previous findings [1, 3–6, 8, 16].

Accumulating literatures have described the difference in lipid profiles between TB and health controls, showing lower levels of total cholesterol, LDL, and high-density lipoprotein (HDL) level in TB cases [30, 31]. Some authors speculated that it might be the result of increased lipid metabolism during inflammatory or infection status for immune response [32]. In our study, however, the levels of LDL and HDL tended to be lower in the new TB group without statistical significance. The reasons for this discrepancy are not clear and may be attributed to the insufficient numbers of TB cases and different patient population in this study.

Based on a survey of symptoms in 313 adult tuberculosis patients, the percentage of cough ≥ 2 weeks, chest pain, anorexia, and body weight loss was reported as only 48.2%, 41.0%, 40.6%, and 44.5%, respectively [33]. In the elderly, one meta-analysis review reported no differences between old and young TB patients with respect to cough, sputum production, weight loss, and fatigue, but older patients had a lower prevalence of fever, sweating, and hemoptysis [34]. In this study, we found that neither a symptom score of greater than 5 points nor any symptom could clearly distinguish TB cases, with the exception of subjective body weight loss. Similarly, the weakness of symptom screening was also demonstrated in a Vietnam study [35]. In fact, we showed that subjective body weight loss is an independent factor associated with pulmonary TB. In this regard, underweight has been shown to be an independent factor associated with tuberculosis [6]. Taken together, elderly diabetic patients with complaints of body weight loss should prompt active TB screening in practice.

This study has several limitations. The first limitation is about the short duration. In an observational study in the U.S., TB was observed to be a seasonal disease, with a peak in spring and trough in late fall [36]. Our study period that covered September to November, the results may thus underestimate of the effect of intensive screening due to relatively lower incidence rate. Secondly, we examined acid fast stain and mycobacterial culture only in suspicious cases. It is likely that we underestimated the occurrence of pulmonary TB in this population. This underestimation may constitute an under-reporting bias and negatively impact the accuracy of the risk factors for TB in this analysis. Further studies are needed to clarify this issue.

## Conclusion

Subjective body weight loss, smoking and liver cirrhosis are independent factors associated with pulmonary tuberculosis in elderly diabetic patients. In these subgroups of patients, active surveillance should be prompted to identify patients who are in need of treatment. Considering the potential benefit of joint management of TB and DM, further investigations are needed to study the impact of active screening program on the treatment outcomes in elderly diabetic patients with TB.

## Abbreviations

ALT: Alanine aminotransferase
BMI: Body mass index
BW: Body weight
CDC: Centers for Disease Control
CGMH: Chang Gung Memorial Hospital
DM: Diabetes mellitus
HbA1c: Glycated hemoglobin
HDL: High-density lipoprotein
IDF: International Diabetes Federation
LDL: Low-density lipoprotein
NAHSIT: Nutrition and Health Survey in Taiwan
TB: Tuberculosis
TG: Triglyceride
WHO: World Health Organization.
